# Systematic review and meta-analysis of rural-urban disparities in Alzheimer’s disease dementia prevalence

**DOI:** 10.1016/j.tjpad.2025.100305

**Published:** 2025-07-25

**Authors:** Abe Mollalo, Mackenzie Kramer, Maxwell Cutty, Benyamin Hoseini

**Affiliations:** a Department of Public Health Sciences, Medical University of South Carolina, Charleston, SC 29425, USA; b College of Computing, Clemson University, Clemson, SC 29634, USA; c Department of Health Sciences and Research, Medical University of South Carolina, Charleston, SC 29425, USA; d Pharmaceutical Research Center, Pharmaceutical Technology Institute, Mashhad University of Medical Sciences, Mashhad, Iran

**Keywords:** Alzheimer’s disease, Rural-urban disparities, Socioeconomics, Meta-analysis

## Abstract

**Background:**

The prevalence of Alzheimer’s disease (AD) dementia varies between rural and urban areas worldwide, with studies reporting mixed patterns. In this study, we conducted a systematic review and meta-analysis to pool the odds ratio (OR) of rural-to-urban prevalence and explored contributing regional and socioeconomic factors.

**Methods:**

We performed comprehensive searches in PubMed, MEDLINE, CINAHL, Web of Science, and Scopus (January 2000-August 2024) for peer-reviewed studies reporting individual-level AD dementia prevalence comparisons between rural and urban settings. A random-effects model was used to calculate pooled OR at a 95 % confidence interval (CI). Prespecified subgroup analyses examined variations by WHO-defined regions, healthcare expenditure, income level, and educational attainment.

**Results:**

The meta-analysis incorporated 19 studies (22 datasets, *N* = 584,863) and found significantly higher AD dementia prevalence in rural areas (pooled OR = 1.247, 95 % CI: 1.059–1.468), with considerable between-study heterogeneity (I^2^=95.5 %). Regional subgroup analyses revealed marked disparities in the Western Pacific (OR = 1.416, 95 % CI: 1.083–1.851) and Southeast Asia (OR = 1.382, 95 % CI: 1.058–1.805), contrasting with nonsignificant findings in the Americas (OR = 0.989, 95 % CI: 0.785–1.247). Socioeconomic stratification showed pronounced rural disadvantages in: (1) lower healthcare expenditure regions (≤7.5 % GDP: OR = 1.268, 95 % CI: 1.043–1.542) and (2) among lower-middle to upper-middle income countries (OR = 1.260, 95 % CI: 1.030–1.542). This disparity attenuated in high-income settings (OR = 1.206, 95 % CI: 0.979–1.486) and in regions with healthcare expenditure >7.5 % GDP (OR = 1.16, 95 % CI: 0.87–1.53). Educational stratification revealed significant rural-urban disparities in regions with lower educational attainment (≤8.1 mean schooling years: OR=1.43, 95 % CI: 1.15–1.79). In contrast, regions with higher educational attainment (>8.1 years) showed no significant difference (OR=1.05, 95 % CI: 0.89–1.25).

**Conclusion:**

This review provides useful evidence that AD dementia prevalence is higher in rural areas than in urban areas, particularly in resource-limited settings. Our findings call for targeted rural interventions in vulnerable regions and further research into how healthcare infrastructure and education jointly influence AD dementia disparities.

## 1 Introduction

Alzheimer’s disease (AD) dementia is an escalating public health problem, ranking as the fifth leading cause of death worldwide[1]. Since 1990, the number of individuals affected by AD dementia has nearly doubled[2], with approximately 50 million living with AD dementia in 2019—a figure projected to exceed 150 million by 2050[3]. This growing prevalence has posed a significant burden on societies and healthcare systems. In 2019, the global economic cost of AD dementia surpassed $1.3 trillion[3], with estimates suggesting this will exceed $2 trillion by 2030[4]. This growing burden, however, is not evenly distributed globally. Low- and middle-income countries (LMICs) are disproportionately affected, and their share of the global dementia population is expected to rise to 63 % by 2030 and 71 % by 2050[5]. The most remarkable growth is expected in North Africa and the Middle East (367 %) and eastern sub-Saharan Africa (357 %)[6].

The prevalence of AD dementia varies between rural and urban areas worldwide, with studies reporting mixed patterns. Some studies suggest that rural areas bear a higher burden due to factors such as limited access to healthcare, shortages of specialized care, and lower educational attainment[7,8]. In particular, access to providers skilled in neuropsychological evaluation is limited in many rural communities, contributing to delays in diagnosis or underdiagnosis of AD dementia. Given the absence of an effective treatment to halt AD dementia progression, prevention through modification of known risk factors remains essential, particularly as nearly half of all dementia cases may be preventable[9]. The 2024 Lancet Commission identifies fourteen modifiable risk factors for AD dementia. Rural populations may be more susceptible to many of these risk factors due to structural disadvantages such as lower educational attainment, higher rates of hypertension, obesity, hearing impairment, smoking, depression, physical inactivity, social isolation, and reduced access to quality healthcare[9]. This increased vulnerability likely contributes to the higher observed prevalence of AD dementia in rural areas.

Conversely, some studies suggest that urban areas are more affected due to higher exposure to ambient air pollution, higher stress levels, and lower exposure to green space[10,11]. It should be noted that while air pollution is typically viewed as an urban issue, rural populations—particularly agricultural workers—can experience acute exposure to fine particulate matter from farming practices, which has been linked to increased dementia risk[12]. Some studies, however, found no significant differences between rural and urban populations[13], highlighting the need for a systematic synthesis of recent evidence.

Although previous studies have reviewed rural-urban disparities in AD dementia[[14], [15], [16]], there has yet to be a recent comprehensive analysis to quantify the differences. Given the mixed findings in previous studies, a meta-analysis is essential to consolidate the existing evidence. Thus, this study aims to systematically assess and quantify rural-urban differences in AD dementia prevalence. We hypothesize that the AD dementia prevalence in rural areas is significantly higher than in urban areas. However, the extent of the disparity may vary by the World Health Organization (WHO)-defined geographic regions, income levels, health expenditure, and educational attainment. Thus, the specific objectives of this study are to 1) provide a pooled estimate of odds ratio (OR) of AD dementia prevalence in rural versus urban areas, 2) examine variations based on the WHO-defined regions, and 3) assess how the association varies by countries’ healthcare expenditure, income, and years of schooling. The findings can provide valuable insights for informing policy interventions and ensuring more effective resource allocation tailored to the specific rural and urban populations.

## 2 Methods

### 2.1 Search strategy

We adhered to the Preferred Reporting Items for Systematic Reviews and Meta-Analyses (PRISMA) guidelines to ensure a rigorous selection of articles for the systematic review and meta-analysis[17]. The PRISMA checklist is available in Appendix 1. A comprehensive search was conducted across five electronic databases: PubMed, MEDLINE, CINAHL, Web of Science, and Scopus. The search included articles published between January 1, 2000, and August 16, 2024, without imposing geographic restrictions. The initial search terms included variations related to the concept of rural (e.g., rural, ruralities, rurality, ruralness, remote, remoteness), urban (e.g., urban, urbanicity, urbanism, urbanity, urbanization, urbanizations, urbanize, urbanized, urbanizes, urbanizing), and Alzheimer’s Disease (e.g., dementia, Alzheimer, ADRD). These terms were combined using logical operators. To ensure comprehensiveness, a manual search of reference lists from relevant articles was also conducted. The retrieved abstracts and titles were imported into Covidence (https://www.covidence.org/) to manage the screening process. This tool automatically identifies and removes duplicate records while enabling independent and blinded article screening by multiple reviewers.

### 2.2 Eligibility criteria

We included peer-reviewed studies that assessed rural-urban disparities in AD dementia if they met all of the following criteria: 1) published in English with full text available; 2) reported dementia prevalence or incidence separately for rural and urban populations (either directly or derivable from data); 3) conducted individual-level data analysis (i.e., ecological studies were excluded); 4) presented original research (reviews, commentaries, conference proceedings, and editorials were excluded). Eligible articles were read, tagged, and summarized.

### 2.3 Data extraction

After finalizing the articles, two independent reviewers (MC and MK) extracted data from each included study using a standardized spreadsheet. Study authors were contacted for additional information if full texts or relevant data were missing. The data extracted were first author, publication year, country, sampling method, and study design. Additionally, for both rural and urban populations, quantitative data were extracted on the mean age of participants, the proportion of male and female participants, education level, and either the prevalence (or incidence) of AD dementia or the number of AD dementia cases and the total population. Given the absence of a universal definition for rurality, the rural and urban classifications used in each study reported by the authors were adopted. Moreover, to assess consistency between reviewers, interrater reliability was calculated using Cohen’s kappa coefficient, which indicated a high level of agreement (κ = 0.87) during both the screening and data extraction stages.

### 2.4 Risk of bias assessment

The risk of bias was evaluated using a Joanna Briggs Institute (JBI) critical appraisal tool[18], which assesses observational studies that report prevalence. The JBI checklist consists of nine questions (e.g., was the sample frame suitable for the target population? was the statistical analysis appropriate?). Each question is rated as “yes,” “no,” “unclear,” or “not applicable.” For scoring, a “yes” response is assigned 1 point, while “no,” “unclear,” and “not applicable” receive 0 points. The total score for each study is expressed as a percentage and categorized into three risk levels: high risk of bias (20–50 % of items scored “yes”), moderate risk of bias (50–80 % of items scored “yes”), and low risk of bias (80–100 % of items scored “yes”). The process described above was performed by two reviewers (MC and MK) independently, and any disagreement was resolved in consultation with a third reviewer (AM or BH).

### 2.5 Meta-analysis

For each study, ORs with 95 % confidence intervals (CIs) were calculated to compare the prevalence of AD dementia in rural versus urban settings, with urban areas serving as the reference group. These estimates were derived from the number of AD dementia cases and the total population in rural and urban areas. A pooled OR with a corresponding 95 % CI was then calculated using meta-analytic techniques. Given the expected variability in study characteristics—particularly in sample sizes and populations—a random-effects model was employed to generate pooled estimates.

Heterogeneity was assessed using I² statistic and Cochran’s Q test. I² values below 25 % were considered indicative of low heterogeneity, values between 25 % and 75 % as moderate, and values above 75 % as high[19]. A Cochran’s Q test p-value of <0.05 was regarded as evidence of statistically significant heterogeneity[20].

Publication bias was assessed visually via funnel plot symmetry and statistically using Egger’s regression test[21]. Egger’s test and funnel plot asymmetry are known to be unreliable when fewer than 10 studies are included in a meta-analysis due to low power and the increased risk of misleading results[22]. In our case, the final included studies in the meta-analysis were sufficient to justify the use of these methods and interpret the results with greater confidence. Duval and Tweedie’s trim-and-fill method was further applied to identify and adjust for any potential missing studies[23].

Sensitivity analyses were conducted using a leave-one-out approach, in which each study was sequentially excluded to examine its impact on the pooled effect size.

Subgroup analyses were conducted to explore potential sources of heterogeneity. First, a subgroup analysis was performed based on the WHO-defined regions (https://data.who.int/countries/). Next, countries were classified into high-income countries (HICs) and low- and middle-income (LMICs) countries using World Bank income level data (https://www.worldbank.org/). Additional subgroup analyses were conducted based on current health expenditure as a percentage of gross domestic product (GDP) using World Bank data. As a proxy for educational attainment, mean years of schooling was used, based on Human Development Reports (https://hdr.undp.org/). When data for specific subgroups—such as different age groups or individuals with primary school education or less—were available, they were pooled to calculate weighted ORs for each subgroup.

To assess potential confounding due to overlapping subgroup characteristics (e.g., income, education, healthcare expenditure, and geographic region), a cross-tabulation of the included studies across these variables was conducted. This descriptive matrix enabled identification of patterns and imbalances in subgroup combinations that could affect interpretation. Additionally, a sensitivity analysis was conducted by excluding the single study classified as both high income and low education, to evaluate its potential influence on the pooled estimates in the low education subgroup.

All statistical analyses were performed using Comprehensive Meta-Analysis software, version 3 (https://www.meta-analysis.com).

## 3 Results

### 3.1 Study selection

**Fig. 1:**
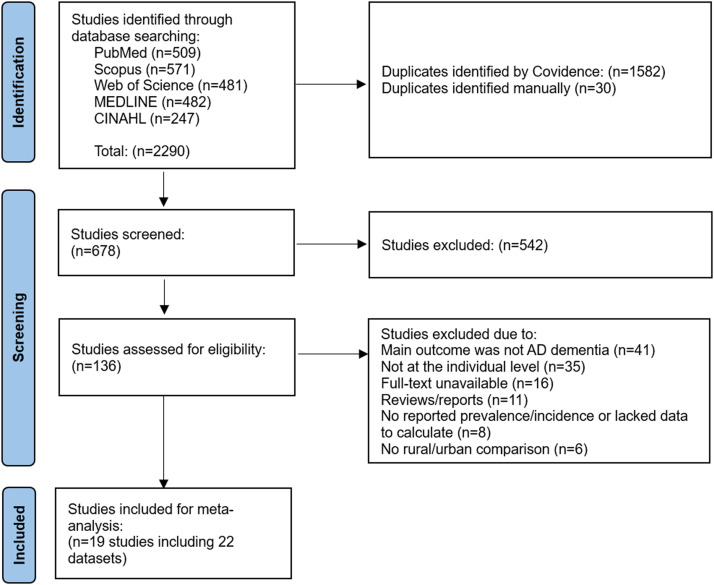
PRISMA flow diagram summarizing the study selection process.

The initial search yielded 2290 articles, which was reduced to 678 after removing duplicates. Of these, 542 articles were excluded after scanning titles and abstracts and determining they were not relevant to our investigation. Of the 136 candidate articles, 117 articles were omitted after full texts screening. The final selection included 19 studies comprising 22 datasets eligible for meta-analysis. Fig. 1 presents the PRISMA flow diagram summarizing the study selection process.

### 3.2 Study characteristics

The geographic distribution of the included datasets spanned multiple regions, with China and India collectively accounting for half of the total datasets (*n* = 11/22, 50.0 %). China had the highest representation (*n* = 8/22, 36.4 %)[24–31], followed by India (*n* = 3/22, 13.6 %)[29,32,33]. United States[34,35] and Taiwan[36,37] each contributed two datasets (*n* = 2/22, 9.1 %), while Bangladesh[38], Canada[39], Egypt[10], Mexico[29], Pakistan[40], Peru[29] and Portugal[41] each accounted for a single dataset (*n* = 1/22, 4.5 %). Almost all articles were limited to a single country except for one[29], which included datasets from four countries (China, India, Mexico and Peru). Almost all studies were conducted in the Northern Hemisphere (*n* = 21/22, 95.5 %), with only one study originating from the Southern Hemisphere[29] (*n* = 1/22, 4.5 %). In terms of continental distribution, the highest proportion of datasets came from Asia (*n* = 15/22, 68.2 %), followed by North America (*n* = 4/22, 18.2 %), with the remaining data from Europe (*n* = 1/22, 4.5 %), Africa (*n* = 1/22, 4.5 %), and South America (*n* = 1/22, 4.5 %). Notably, no studies were conducted in Oceania. Based on the World Bank classification, most studies were conducted in upper-middle-income countries (*n* = 11/22, 50.0 %) and high-income countries (*n* = 6/22, 27.3 %), while fewer studies were conducted in lower-middle-income countries (*n* = 5/22, 22.7 %).

Rural-urban classification varied across studies. Among the 19 included studies, the majority (*n* = 12/19, 63.2 %) did not provide a clear definition of rurality. Among the studies that did define rurality (*n* = 7/19, 36.8 %), various classification methods were employed, including population thresholds (e.g., <2500 for rural [*n* = 1/7, 14.3 %] or <500,000 for rural [*n* = 1/7, 14.3 %]), census-based population density criteria (*n* = 2/7, 28.6 %), administrative jurisdictions (*n* = 1/7, 14.3 %), postal codes (*n* = 1/7, 14.3 %), and urbanization levels (*n* = 1/7, 14.3 %). Most of the included studies (*n* = 17/19, 89.5 %) employed a cross-sectional design, while one study utilized a case-control approach (*n* = 1/19, 5.3 %) and another combined cross-sectional and cohort study designs (*n* = 1/19, 5.3 %).

Among the 19 included studies, multistage sampling was the most common approach (*n* = 8/19, 42.1 %), which included methods such as multistage stratified cluster sampling (*n* = 3/19, 15.8 %), multistage stratified random sampling (*n* = 2/19, 10.5 %), multistage clustered random sampling (*n* = 2/19, 10.5 %), and multistage stratified probability proportional-to-size sampling (*n* = 1/19, 5.3 %). Consecutive sampling from a secondary source was employed in three studies (*n* = 3/19, 15.8 %). Stratified sampling (*n* = 1/19, 5.3 %), stratified consecutive sampling (*n* = 1/19, 5.3 %), and stratified simple random sampling (*n* = 2/19, 10.5 %) were also used. Random sampling appeared in two studies (*n* = 2/19, 10.5 %), while convenience sampling (*n* = 1/19, 5.3 %) and catchment area sampling (*n* = 1/19, 5.3 %) were each used in a single study. Table 1 presents the main characteristics of the included studies. The risk of bias assessment classified most studies (*n* = 11/19, 57.9 %) as low risk, followed by moderate risk (*n* = 7/19, 36.8 %), and high risk (*n* = 1/19, 5.3 %) (Table 1). An item-by-item analysis of the JBI checklist revealed that most studies had strong methodological quality in several key areas: 18 out of 19 studies had an appropriate sample frame, and all employed appropriate statistical analyses. Eighteen studies also had sufficient data coverage, and 17 used reliable measurement methods. However, larger variability was observed in other domains: only 14 studies clearly reported an appropriate sampling method, 13 had adequate sample sizes, and 15 used validated approaches for condition identification. The most frequent limitation was inadequate reporting or handling of response rates, with only 9 studies meeting this criterion. A detailed breakdown of the scores for each JBI checklist item is available in Appendix 2.

**Table 1:** Main characteristics of the included studies.

No.	Author	Year	Country	Studyd esign	Sample size		Rural-urban classification	Mean Age	Females (%)	Primary school or less (%)	Risk of bias
					Rural	Urban					
1	Chen[24]	2012	China	Cross-Sectional	1181	1736	NA	73.36	52.86	58.18	Low
2	Ding[25]	2020	China	Cross-Sectional	246,369	108,490	Jurisdictions	69.68	51.53	NA	Low
3	Drummond[39]	2016	Canada	Cross-Sectional	14,438	42,940	Postal Code	75.51	57.14	NA	Low
4	Hall[34]	2000	US	Cross-Sectional	79	144	Population-based	77.29	60.54	21.48	Moderate
5	Hu[26]	2022	China	Cross-Sectional	9778	7811	NA	71.7	54.78	64	Low
6	Jia[27]	2014	China	Cross-Sectional	4180	6096	NA	70.96	57.39	65.25	Low
7	Jia[28]	2020	China	Cross-Sectional	16,854	19,266	Population-based	70.26	50.30	50.3	Low
8	Khedr[10]	2015	Egypt	Cross-Sectional	367	324	NA	66.17	50.80	NA	Moderate
9	Lee[32]	2023	India	Cross-Sectional	20,721	10,756	NA	69.24	52.01	77.69	Low
10	Liu[36]	2019	Taiwan	Case-Control	6414	6737	Population Density	79.45	59.11	NA	Low
11	Liu[37]	2022	Taiwan	Cross-Sectional	4115	2624	Urbanization levels	76.24	52.32	77.37	Moderate
12	Naheed[38]	2023	Bangladesh	Cross-Sectional	1899	896	NA	67	51.02	65.7	Moderate
13	Noreen[40]	2018	Pakistan	Cross-Sectional	307	513	NA	60.7	54.55	53.78	High
14	Nunes[41]	2010	Portugal	Cross-Sectional	713	433	NA	66.34	59.69	73.64	Moderate
15	Raina[33]	2014	India	Cross-Sectional	500	500	NA	68.98	49.1	37.2	Moderate
16	Wang[30]	2022	China	Cross-Sectional	362	411	NA	73.28	53.56	52.26	Moderate
17	Weden[35]	2018	US	Cross-Sectional	3522	19,374	Population Density	68.12	54.35	19.01	Low
18	Zhao[31]	2010	China	Cross-Sectional& Cohort	4876	11,036	NA	68.2	54.85	NA	Low
19	Rodriguez[29]	2008	China	Cross-Sectional	1002	1160	NA	73.1	56.29	49.86	Low
			India		999	1005		72.7	55.69	75.70	
			Mexico		1000	1002		74.1	63.33	70.83	
			Peru		552	1381		74.2	61.20	18.21	

### 3.3 Descriptive statistics

The number of study participants in the included studies ranged from 223 to 354,859, with a total of 584,863 participants (mean = 26,585, SD = 74,773.3). The mean differences in AD dementia cases between rural and urban groups were not significant (*P* > 0.05). Similarly, the difference in the number of participants between rural and urban groups was not significant (*P* > 0.05). When stratified by rural and urban settings, the number of AD dementia cases in rural areas ranged from 7 to 3076, totaling 11,334 cases (mean = 515.2, SD = 770.6), whereas urban areas reported between 16 and 3388 cases, with a total of 11,860 cases (mean = 539.1, SD = 962.7). Additionally, the number of participants in rural areas ranged from 79 to 246,369, totaling 340,228 participants (mean = 15,464.9, SD = 51,904.09), while urban areas included between 144 and 108,490 participants, with a total of 244,635 participants (mean = 11,119.8, SD = 23,942.6).

**Fig. 2:**
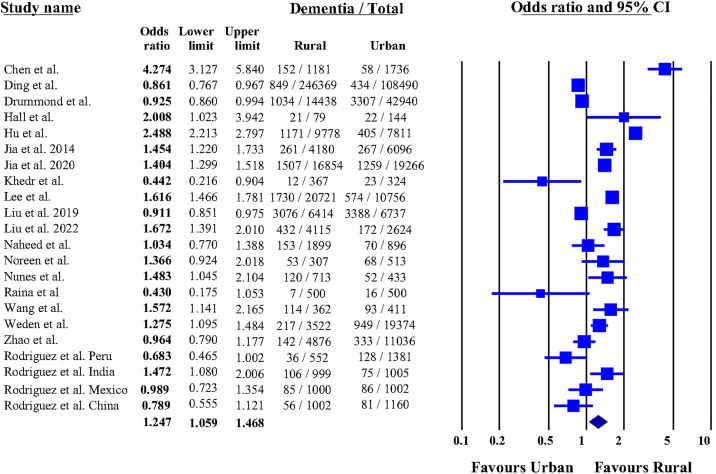
Forest plot of the meta-analysis on rural-urban disparities in AD dementia prevalence. Studies with OR > 1 suggest a higher prevalence of AD dementia in rural areas compared to urban areas. The diamond data marker represents the pooled OR estimate.

The point estimates of ORs for AD dementia prevalence, using urban as the reference, ranged from 0.43 to 4.27 (mean = 1.36, SD = 0.82). Fig. 2 presents the forest plot of individual study ORs with their corresponding 95% CIs. Among the 22 datasets, half (*n* = 11/22, 50.0 %) reported higher ORs of AD dementia prevalence in rural areas compared to urban areas, while a few studies (*n* = 4/22, 18.2 %) found significantly higher ORs in urban areas. The remaining studies (*n* = 7/22, 31.8 %) observed no significant difference in ORs of AD dementia prevalence between rural and urban areas.

The results of the meta-analysis under the random-effects model indicate that the pooled OR of AD dementia prevalence was higher among rural areas than in urban areas [OR = 1.247, 95 % CI: 1.059–1.468; *P* < 0.05] (Fig. 2). The fixed-effects model also confirmed higher ORs in rural areas with a pooled OR of 1.197 (95 % CI: 1.161–1.235). Substantial heterogeneity was observed among the included studies (*Q* = 466.88, *P* < 0.001, I² = 95.5 %).

### 3.4 Publication bias assessment and sensitivity analysis

**Fig. 3:**
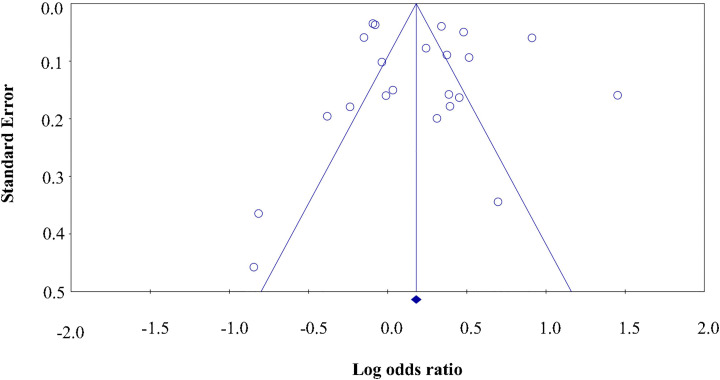
Funnel plot assessing publication bias in the included studies. The symmetrical distribution of studies indicates a low likelihood of publication bias.

Multiple methods were employed to evaluate potential publication bias. Visual inspection of the funnel plot (Fig. 3) revealed no substantial asymmetry, suggesting a low likelihood of publication bias. This was further supported by Egger’s regression test, which indicated no significant small-study effects (*t* = 0.62, *P* = 0.54). Additionally, the trim-and-fill method did not impute any missing studies, and the adjusted pooled estimates remained identical to the original values, reinforcing the robustness of the findings.

Sensitivity analyses stratified by risk of bias revealed consistent patterns: low-risk studies showed significantly higher rural prevalence (OR=1.26, 95 % CI: 1.04–1.54; I²=97 %), while high/moderate-risk studies showed non-significant but directionally similar effects (OR=1.23, 95 % CI: 0.95–1.61; I²=73 %). Moreover, a leave-one-out sensitivity analysis was conducted to assess the influence of individual studies on the overall pooled estimate. The pooled OR remained statistically significant across all iterations, ranging from 1.179 (95 % CI: 1.007–1.381) to 1.283 (95 % CI: 1.088–1.513), indicating the robustness of the results. The heterogeneity estimates (I²) consistently exceeded 93 % in all scenarios, suggesting that no single study was solely responsible for the observed heterogeneity. The exclusion of one article[26] led to the largest reduction in heterogeneity (I² = 93.46 %), yet the overall effect size remained stable, further supporting the reliability of the results. It should be noted that among all included studies, only one study reported incidence rather than prevalence[36]. The leave-one-out sensitivity analysis showed that excluding this study did not systematically change the overall results, supporting the robustness of the pooled estimate. Appendix 3 provides a detailed summary of the sensitivity analysis results, including the pooled ORs and heterogeneity estimates for each study exclusion.

### 3.5 Subgroup analysis

#### 3.5.1 WHO-defined regions

Subgroup analyses examined rural-urban disparities in AD dementia prevalence across WHO regions, including the Western Pacific, Americas, and Southeast Asia (Table 2). In the Western Pacific region, the meta-analysis yielded a pooled OR of 1.416 (95 % CI: 1.083–1.851), indicating a significantly higher OR of AD dementia prevalence in rural areas compared to urban areas, with substantial heterogeneity observed (*Q* = 349.8, *P* < 0.001; I² = 97.43 %). Similarly, in Southeast Asia, the pooled OR was 1.382 (95 % CI: 1.058–1.805), also suggesting a significantly higher OR of AD dementia prevalence in rural areas compared to urban areas, with moderate to high heterogeneity across studies (*Q* = 8.04, *P* < 0.001; I² = 75.12 %). However, in the Americas region, the pooled OR was 0.989 (95 % CI: 0.785–1.247), indicating no significant difference in AD dementia prevalence between rural and urban areas, though heterogeneity remained substantial (*Q* = 24.93, *P* < 0.001; I² = 79.94 %).

**Table 2:** The subgroup analyses rural-urban disparities in AD dementia prevalence by WHO region, years of schooling, healthcare expenditure, and income.

Subgroup	Pooled OR (95 % CI)	Heterogeneity (Q, p-value)	I²
**WHO Region**			
Western Pacific	1.416 (1.083–1.851)	*Q* = 349.8, *P* < 0.001	97.43 %
Southeast Asia	1.382 (1.058–1.805)	*Q* = 8.04, *P* < 0.001	75.12 %
Americas	0.989 (0.785–1.247)	*Q* = 24.93, *P* < 0.001	79.94 %
**Years of Schooling**			
≤ 8.1 years	1.434 (1.147–1.791)	*Q* = 243.72, *P* < 0.001	95.49 %
> 8.1 years	1.054 (0.887–1.252)	*Q* = 71.9, *P* < 0.001	87.48 %
**Healthcare Expenditure**			
≤ 7.5 % of GDP	1.268 (1.043–1.542)	*Q* = 398.95, *P* < 0.001	95.99 %
> 7.5 % of GDP	1.156 (0.871–1.533)	*Q* = 26.66, *P* < 0.001	84.99 %
**Income**			
Lower-middle/Upper-middle income	1.260 (1.030–1.542)	*Q* = 275.11, *P* < 0.001	94.9 %
High-income	1.206 (0.979–1.486)	*Q* = 60.66, *P* < 0.001	90.1 %

#### 3.5.2 Years of schooling

Subgroup analyses were conducted based on the mean years of schooling as a proxy for educational attainment (Table 2). Studies were stratified into two subgroups: regions with a mean schooling duration of ≤8.1 years and those with >8.1 years. Educational stratification revealed significant rural-urban disparities in regions with lower educational attainment (≤8.1 mean schooling years: OR=1.43, 95 % CI: 1.15–1.79), with extreme heterogeneity (I²=95.5 %, *Q* = 243.72, *p* < 0.001). In contrast, regions with higher educational attainment (>8.1 years) showed no significant difference (OR=1.05, 95 % CI: 0.89–1.25), though substantial heterogeneity persisted (I²=87.5 %, *Q* = 71.9, *p* < 0.001). Although heterogeneity in higher educational attainment regions remained high (*Q* = 71.9, *P* < 0.001; I² = 87.48 %), it was slightly lower than in the lower years of schooling subgroup.

#### 3.5.3 Healthcare expenditure

Subgroup analyses were conducted based on healthcare expenditure (% of GDP), with studies categorized into low and middle (≤7.5 %) and high (>7.5 %) expenditure groups (Table 2). In regions with lower healthcare spending (≤7.5 %), the meta-analysis yielded a pooled OR of 1.268 (95 % CI: 1.043–1.542), indicating significantly higher OR of AD dementia prevalence in rural areas compared to urban areas, with substantial heterogeneity (*Q* = 398.95, *P* < 0.001; I² = 95.99 %). In contrast, in regions with higher healthcare expenditure (>7.5 %), the pooled OR was 1.156 (95 % CI: 0.871–1.533), suggesting no significant rural-urban differences in AD dementia prevalence. Although heterogeneity remained high in this group (*Q* = 26.66, *p* < 0.001; I² = 84.99 %), it was slightly lower than in the low and middle expenditure subgroup.

#### 3.5.4 Income

Subgroup analyses based on World Bank income levels were conducted, with studies categorized into lower-middle and, upper-middle income, and high-income regions (Table 2). In lower-middle and upper-middle income regions, the meta-analysis yielded a pooled OR of 1.260 (95 % CI: 1.030–1.542), indicating significantly higher ORs of AD dementia prevalence in rural areas compared to urban areas, with high heterogeneity observed (*Q* = 275.11, *P* < 0.001; I² = 94.9 %). In contrast, the pooled OR in high-income regions was 1.206 (95 % CI: 0.979–1.486), showing no significant difference in AD dementia prevalence between rural and urban areas. However, a substantial heterogeneity was still observed (*Q* = 60.66, *P* < 0.001, I² = 90.1 %).

To examine potential overlap between subgroup variables, we cross-tabulated studies by education, income level, healthcare expenditure, and WHO region (Appendix 4, Table S1). The findings revealed that several subgroup categories were not independent. For instance, most low-education studies were from lower-income or lower-healthcare-expenditure settings. Notably, only one study fell into the high-income and low-education category. A sensitivity analysis excluding this study yielded a pooled OR of 1.423 (95 % CI: 1.125–1.800) in the low education subgroup (Appendix 4, Table S2), nearly identical to the original estimate (OR = 1.434, 95 % CI: 1.147–1.791), with similarly high heterogeneity (I² = 95.9 %), suggesting that the main finding in this subgroup is robust and not driven by this potentially confounding study.

## 4 Discussion

### 4.1 Study design and findings

Given the inconsistent global evidence on rural–urban differences in AD dementia prevalence, we conducted an updated meta-analysis of individual-level studies comparing prevalence across these settings. Our findings revealed that rural areas face significantly higher odds of AD dementia, a disparity likely driven by a heavier burden of modifiable risk factors and reduced access to healthcare. Effective prevention strategies in rural areas should prioritize cardiovascular health, smoking cessation, physical activity, social engagement, sensory care, and improved access to both primary and specialist services. Community-based education and telehealth may also help address structural barriers. Importantly, the disparity was most pronounced in LMICs and in regions with lower healthcare spending, suggesting that socioeconomic development and healthcare quality are critical contributors. Without targeted, context-specific interventions, these rural–urban gaps are likely to widen alongside the growing global burden of AD dementia.

Rural areas often face critical gaps in healthcare infrastructure, including limited access to specialists trained in neuropsychological evaluation, which can lead to delayed or missed dementia diagnoses. Studies by Xu et al. (2022)[42] and Kosteniuk et al. (2024)[43] highlight lower rates of early diagnosis and reduced use of health services in rural populations—both before and after an AD dementia diagnosis. These systemic shortcomings underscore the urgent need to expand diagnostic and treatment services in underserved communities. However, improving detection alone is insufficient; rural residents frequently lack access to follow-up care, disease management, and preventive services. Without trained providers and the infrastructure to support care planning and behavioral interventions, earlier diagnosis may offer limited benefit. Effective dementia prevention in rural settings must therefore go beyond identification to include robust education, adequate resources, and support systems that enable sustained risk reduction and continuity of care.

While several meta-analyses have examined global AD dementia rates[5,44], direct rural-urban comparisons remain limited and show conflicting results. For instance, Arsenault-Lapierre et al. (2023)[16] found that individuals in rural areas experienced higher mortality rates, more frequent hospitalizations, and less access to specialized care than in urban areas. However, their review spanned less than two years and did not include a quantitative assessment of the significance of the findings. Earlier, Russ et al. (2012)[45] found no significant difference in dementia risk between rural and urban populations, regardless of diagnostic subtype. However, their review included outdated studies (pre-2000), conducted before recent urbanization patterns emerged[46]. In contrast, our study reflects more recent data and suggests that this difference in pooled prevalence estimates may, in part, reflect the uneven pace of urbanization and development over the past two decades in the regions where these studies were conducted.

Subgroup analyses showed consistent rural-urban disparities across healthcare expenditure and income levels, with significantly higher AD dementia prevalence in rural areas of lower-resource regions. This likely reflects systemic barriers including (1) limited access to preventive care and cognitive screening, (2) shortages of dementia specialists, including neurologists and geriatricians, and (3) inadequate access to diagnostic imaging technologies such as magnetic resonance imaging and positron emission tomography scans—all of which contribute to delayed diagnosis at more advanced disease stages. These findings align with Wang et al. (2016) who demonstrated widening rural-urban healthcare expenditure gaps in China (rural: 4.9–8.9 % vs urban: 4.4–6.0 % of total consumption)[47], and Ying et al. (2020), who reported higher out-of-pocket healthcare burdens in rural populations[48].

Our analysis found no significant rural-urban differences in AD dementia prevalence in regions with higher healthcare expenditure or income levels. This aligns with the findings from Lee et al. (2014), who reported no significant rural-urban differences in total health expenditures in the US[49], and a large cohort study of Medicare beneficiaries, which found comparable healthcare utilization across rural and metropolitan counties[15]. Together, these results suggest that adequate healthcare access and resource availability may help reduce or eliminate rural-urban disparities in AD dementia prevalence.

Subgroup analyses suggested a protective role of education against rural-urban disparities, potentially supporting the cognitive reserve hypothesis[50,51]; however, this effect is likely confounded by overlapping socioeconomic factors. Notably, 92 % of low-education studies were also from lower-income regions with low healthcare expenditure, indicating that the observed education effect may reflect broader socioeconomic disadvantage rather than the impact of education alone. Similarly, high healthcare expenditure was exclusive to studies from the Americas, conflating regional healthcare systems with spending effects. Crimmins et al. (2018) also observed that higher education is associated with longer cognitively healthy life[52], though this may stem from the combined benefits of education, income, and healthcare access.

### 4.2 Data limitations and generalizability

Our meta-analysis exhibited substantial heterogeneity, prompting the use of a random-effects model to account for true variations across studies. This heterogeneity likely stemmed from differences in sampling methods, study designs, demographic characteristics, and the lack of a standardized definition for rurality. Additionally, variations in study populations, with some cohorts not fully representative of broader rural-urban population and the inclusion of smaller studies, may have further contributed to the observed variability. Despite these factors, leave-one-out sensitivity analyses confirmed that no single study unduly influenced the pooled estimates, and there was no evidence of systematic bias linked to study quality. Notably, the inclusion of one incidence-based study (as opposed to prevalence-based study) did not significantly alter the overall estimates. Stratified analyses by risk of bias revealed that studies deemed low-risk reported significantly higher rural prevalence, while high- and moderate-risk studies showed directionally consistent but non-significant effects. This pattern suggests that rural disadvantage in AD dementia prevalence is robust and that lower-quality studies may underestimate its magnitude. Publication bias was not a major concern; visual inspection of funnel plot, Egger’s regression test, and Duval and Tweedie’s trim-and-fill analysis indicated no significant small-study effects or missing studies.

Beyond the sources of heterogeneity, several other limitations can affect the generalizability of our findings. The predominance of cross-sectional studies restricts our ability to draw causal inferences about rural–urban differences. Moreover, our dataset included studies from only 11 countries—primarily in the Northern Hemisphere—thereby limiting global representation and potentially skewing results toward region-specific patterns in healthcare systems and rural classifications. This geographic concentration underscores the need for more research in underrepresented regions, particularly in the Global South, to ensure equitable insights into AD dementia prevalence disparities. We also restricted our search to English-language publications, which may introduce language bias; however, prior evidence suggests that such restrictions do not systematically distort findings in medical research[53,54].

Another key limitation lies in the confounding among subgroup variables. Factors such as education, income, and healthcare expenditure often cluster regionally and are highly correlated, complicating the interpretation of subgroup effects. While we used descriptive cross-tabulations and targeted sensitivity analyses to explore these relationships, insufficient granularity in the available data precluded formal meta-regression to adjust for these overlapping factors. Additionally, inconsistent definitions of rural and urban areas across studies pose further challenges. Variations in classification criteria and geographic thresholds may lead to misclassification bias, reduce the comparability of prevalence estimates, and undermine the validity of subgroup analyses. To address these challenges, future research should adopt standardized definitions of rurality and report key covariates with greater consistency to enable more nuanced and robust subgroup analyses.

Despite these limitations, our review employed a comprehensive and rigorous methodology. We used broad search terms and relevant synonyms to maximize literature coverage, with screening performed by two independent reviewers to minimize the likelihood of omitting key studies or data. These efforts strengthen the reliability of our conclusions and provide a solid foundation for future works in this area.

### 4.3 Future directions

Future research should prioritize high-quality, longitudinal studies with larger and more diverse rural and urban populations, particularly from underrepresented regions like Europe, South America, Africa, and Oceania, to improve the reliability and generalizability of findings. Although current evidence suggests higher AD dementia prevalence in rural areas, early-life exposure to rural environments may have an even greater effect due to prolonged cumulative exposure to environmental and socioeconomic risk factors[45]. Further cohort studies are necessary to determine the role of early-life exposure on the risk of developing AD dementia over time. Moreover, incorporating measures of rural-urban residence into longitudinal studies of AD dementia can provide useful insights into understanding how rural-to-urban migration patterns and life course exposures influence AD dementia disparities. While sensitivity analyses excluding highly confounded cases yielded robust results, residual confounding remains due to the ecological design of meta-analytic subgroups. Future research should stratify by multiple intersecting variables—such as education within income groups—to better isolate their independent effects.

## 5 Conclusion

This systematic review and meta-analysis found that AD dementia prevalence is significantly higher in rural areas compared to urban areas. These disparities were most pronounced in regions with lower income and healthcare investment, suggesting that socioeconomic and healthcare-related factors are major drivers. In contrast, no significant differences were observed in high-income or high-expenditure settings, indicating that equitable healthcare access may narrow the rural–urban gap. Education also appeared to mitigate rural disparities, though its effects were intertwined with income and healthcare access. Our findings imply that rural–urban disparities in AD dementia are not inevitable—they might be shaped by modifiable factors such as healthcare infrastructure, socioeconomic conditions, and educational opportunities. To address these disparities, policy interventions should focus on integrated strategies that combine improved access to education, healthcare, and coordinated preventive services in disadvantaged rural regions. One-dimensional solutions are unlikely to be effective.

## Consent statement

Consent was not necessary.

## Declaration of generative AI and AI-assisted technologies in the writing process

Generative AI was used to assist with grammar editing.

## Funding

AM is supported by the South Carolina SmartState Endowed Center for Environmental and Biomedical Panomics.

## CRediT authorship contribution statement

**Abe Mollalo:** Writing – review & editing, Writing – original draft, Supervision, Methodology, Funding acquisition, Formal analysis, Conceptualization. **Mackenzie Kramer:** Writing – review & editing, Data curation. **Maxwell Cutty:** Writing – review & editing, Data curation. **Benyamin Hoseini:** Writing – review & editing, Writing – original draft, Formal analysis, Conceptualization.

## Declaration of competing interest

Abe Mollalo reports financial support was provided by South Carolina SmartState Endowed Center for Environmental and Biomedical Panomics. Abe Mollalo reports a relationship with South Carolina SmartState Endowed Center for Environmental and Biomedical Panomics that includes: funding grants. Other authors declare that they have no known competing financial interests or personal relationships that could have appeared to influence the work reported in this paper.

## Appendix Supplementary materials

Image, application 1

Image, application 2

Image, application 3

Image, application 4
