# Supplementary material for: Systematic review and meta-analysis of rural-urban disparities in Alzheimer’s disease dementia prevalence
Source: J Prev Alzheimers Dis. 2025 Jul 25;12(9):100305. doi: 10.1016/j.tjpad.2025.100305 (PMC12501328; doi:10.1016/j.tjpad.2025.100305)
Supplement: Supplementary file 2 [file mmc2.docx]

**Appendix 2.** The risk of bias assessment

|  | Chen | Ding | Drummond | Hall | Hu | Jia 2014 | Jia 2020 | Khedr | Lee | Liu 2019 | Liu 2022 | Naheed | Noreen | Nunes | Raina | Wang | Weden | Zhao | Rodriguez |
| --- | --- | --- | --- | --- | --- | --- | --- | --- | --- | --- | --- | --- | --- | --- | --- | --- | --- | --- | --- |
| 1. Sample frame appropriate | Yes | Yes | Yes | Yes | Yes | Yes | Yes | Yes | Yes | Yes | Yes | Yes |  | Yes | Yes | Yes | Yes | Yes | Yes |
| 2. Sampling method appropriate | Yes | Yes | Yes | Yes | Yes |  | Yes |  | Yes | Yes |  | Yes |  | Yes | Yes |  | Yes | Yes | Yes |
| 3. Sample size adequate | Yes | Yes | Yes |  | Yes | Yes | Yes |  | Yes | Yes | Yes | Yes |  |  |  |  | Yes | Yes | Yes |
| 4. Subjects and setting described | Yes |  | Yes | Yes | Yes | Yes | Yes | Yes | Yes | Yes | Yes | Yes | Yes | Yes | Yes | Yes | Yes | Yes | Yes |
| 5. Data analysis coverage sufficient | Yes | Yes | Yes | Yes | Yes | Yes | Yes | Yes | Yes | Yes | Yes | Yes |  | Yes | Yes | Yes | Yes | Yes | Yes |
| 6. Valid condition identification | Yes | Yes | Yes | Yes | Yes | Yes | Yes | Yes | Yes |  | Yes |  | Yes | Yes |  | Yes |  | Yes | Yes |
| 7. Reliable measurement | Yes | Yes |  | Yes | Yes | Yes | Yes | Yes | Yes | Yes | Yes |  | Yes | Yes | Yes | Yes | Yes | Yes | Yes |
| 8. Appropriate statistical analysis | Yes | Yes | Yes | Yes | Yes | Yes | Yes | Yes | Yes | Yes | Yes | Yes | Yes | Yes | Yes | Yes | Yes | Yes | Yes |
| 9. Response rate adequate |  | Yes | Yes |  |  | Yes | Yes |  | Yes | Yes |  | Yes |  |  |  | Yes | Yes |  |  |
| Risk of Bias | Low | Low | Low | Mod | Low | Low | Low | Mod | Low | Low | Mod | Mod | High | Mod | Mod | Mod | Low | Low | Low |
