# Supplementary material for: Systematic review and meta-analysis of rural-urban disparities in Alzheimer’s disease dementia prevalence
Source: J Prev Alzheimers Dis. 2025 Jul 25;12(9):100305. doi: 10.1016/j.tjpad.2025.100305 (PMC12501328; doi:10.1016/j.tjpad.2025.100305)
Supplement: Supplementary file 3 [file mmc3.docx]

**Appendix 3.** The results of the leave-one-out sensitivity analysis

| **Study removed** | **Pooled OR** | **Heterogeneity testing** |
| --- | --- | --- |
| Chen et al. | 1.179 (95% CI: 1.007–1.381) | (**Q** = 402.48, **p** < 0.001, **I²** = 95.03%) |
| Ding et al. | 1.273 (95% CI: 1.074–1.507) | (**Q** = 433.35, **p** < 0.001, **I²** = 95.38%) |
| Drummond et al. | 1.266 (95% CI: 1.064–1.506) | (**Q** = 407.1, **p** < 0.001, **I²** = 95.09%) |
| Hall et al. | 1.23 (95% CI: 1.042–1.452) | (**Q** = 464.62, **p** < 0.001, **I²** = 95.69%) |
| Hu et al. | 1.206 (95% CI: 1.043–1.393) | (**Q** = 305.96, **p** < 0.001, **I²** = 93.46%) |
| Jia et al. 2014 | 1.236 (95% CI: 1.042–1.465) | (**Q** = 462.01, **p** < 0.001, **I²** = 95.67%) |
| Jia et al. 2020 | 1.235 (95% CI: 1.032–1.476) | (**Q** = 447.71, **p** < 0.001, **I²** = 95.53%) |
| Khedr et al. | 1.283 (95% CI: 1.088–1.513) | (**Q** = 459.4, **p** < 0.001, **I²** = 95.65%) |
| Lee et al. | 1.228 (95% CI: 1.035–1.456) | (**Q** = 426.4, **p** < 0.001, **I²** = 95.31%) |
| Liu et al. 2019 | 1.268 (95% CI: 1.068–1.506) | (**Q** = 389.63, **p** < 0.001, **I²** = 94.87%) |
| Liu et al. 2022 | 1.227 (95% CI: 1.037–1.452) | (**Q** = 453.84, **p** < 0.001, **I²** = 95.59%) |
| Naheed et al. | 1.258 (95% CI: 1.063–1.489) | (**Q** = 465.91, **p** < 0.001, **I²** = 95.71%) |
| Nunes et al. | 1.237 (95% CI: 1.046–1.463) | (**Q** = 465.43, **p** < 0.001, **I²** = 95.7%) |
| Rhew et al. | 1.275 (95% CI: 1.082–1.504) | (**Q** = 461.85, **p** < 0.001, **I²** = 95.67%) |
| Rodriguez et al. China | 1.273 (95% CI: 1.077–1.505) | (**Q** = 461.41, **p** < 0.001, **I²** = 95.66%) |
| Rodriguez et al. India | 1.237 (95% CI: 1.045–1.463) | (**Q** = 461.41, **p** < 0.001, **I²** = 95.66%) |
| Rodriguez et al. Mexico | 1.260 (95% CI: 1.065–1.491) | (**Q** = 465.44, **p** < 0.001, **I²** = 95.7%) |
| Rodriguez et al. Peru | 1.280 (95% CI: 1.084–1.512) | (**Q** = 458.6, **p** < 0.001, **I²** = 95.64%) |
| Wang et al. | 1.233 (95% CI: 1.042–1.459) | (**Q** = 464.8, **p** < 0.001, **I²** = 95.7%) |
| Weden et al. | 1.244 (95% CI: 1.047–1.477) | (**Q** = 466.2, **p** < 0.001, **I²** = 95.7%) |
| Zhao et al. | 1.263 (95% CI: 1.066–1.497) | (**Q** = 462.23, **p** < 0.001, **I²** = 95.7%) |
