# Supplementary material for: Systematic review and meta-analysis of rural-urban disparities in Alzheimer’s disease dementia prevalence
Source: J Prev Alzheimers Dis. 2025 Jul 25;12(9):100305. doi: 10.1016/j.tjpad.2025.100305 (PMC12501328; doi:10.1016/j.tjpad.2025.100305)
Supplement: Supplementary file 4 [file mmc4.docx]

**Appendix 4 Table S1. Cross-tabulation of included studies by subgroup variables.** Distribution of studies across combinations of key subgroup variables: education level (high vs. low), income level (high vs. lower-/upper-middle income), healthcare expenditure (high vs. low/medium), and WHO regions (Western Pacific, Americas, Southeast Asia).

**A. Education × Income**

| **Income Level** | **High Education** | **Low Education** | **Total** |
| --- | --- | --- | --- |
| High income | 6 | 1 | 7 |
| Lower-/Upper-middle income | 4 | 11 | 15 |
| **Total** | **10** | **12** | **22** |

**B. Income × Healthcare Expenditure**

| **Income Level** | **High Health Expenditure** | **Low/Medium Health Expenditure** | **Total** |
| --- | --- | --- | --- |
| High income | 4 | 3 | 7 |
| Lower-/Upper-middle income | 1 | 14 | 15 |
| **Total** | **5** | **17** | **22** |

**C. Education × Healthcare Expenditure**

| **Education Level** | **High Health Expenditure** | **Low/Medium Health Expenditure** | **Total** |
| --- | --- | --- | --- |
| High education | 5 | 5 | 10 |
| Low education | 0 | 12 | 12 |
| **Total** | **5** | **17** | **22** |

**D. Region × Income**

| **WHO Region** | **High Income** | **Lower-/Upper-middle Income** | **Total** |
| --- | --- | --- | --- |
| Western Pacific | 3 | 7 | 10 |
| Americas | 3 | 3 | 6 |
| Southeast Asia | 0 | 3 | 3 |
| **Total** | **6** | **13** | **19** |

**E. Region × Healthcare Expenditure**

| **WHO Region** | **High Health Expenditure** | **Low/Medium Health Expenditure** | **Total** |
| --- | --- | --- | --- |
| Western Pacific | 0 | 10 | 10 |
| Americas | 4 | 2 | 6 |
| Southeast Asia | 0 | 3 | 3 |
| **Total** | **4** | **15** | **19** |

**F. Region × Education**

| **WHO Region** | **High Education** | **Low Education** | **Total** |
| --- | --- | --- | --- |
| Western Pacific | 2 | 8 | 10 |
| Americas | 6 | 0 | 6 |
| Southeast Asia | 0 | 3 | 3 |
| **Total** | **8** | **11** | **19** |

**Appendix 4 Table S2.** Study distribution by overlapping Subgroups and pooled effect sizes for overlapping subgroup combinations.

| **Subgroup Combination** | **n/N (%)** | **Pooled OR (95% CI)** |
| --- | --- | --- |
| Low education + Low income | 11/12 (92%) | 1.42 (1.25–1.80) |
| Low education + Low expenditure | 12/12 (100%) | 1.43 (1.147–1.791) |
| High income + High expenditure | 4/5 (80%) | 1.156 (0.87–1.53) |
